# Long-term prognosis after resection of cryptogenic hepatocellular carcinoma

**DOI:** 10.1186/s12893-015-0099-9

**Published:** 2015-10-17

**Authors:** Yu Ohkura, Kazunari Sasaki, Masamichi Matsuda, Masaji Hashimoto, Goro Watanabe

**Affiliations:** Departments of Gastroenterological Surgery, Hepato Pancreato Billiary Surgery Unit, Toranomon Hospital, 2-2-2 Toranomon, Minato-ku, Tokyo 105-8470 Japan

**Keywords:** Cryptogenic hepatocellular carcinoma, Non-B, non-C hepatocellular carcinoma, Long-term prognosis

## Abstract

**Background:**

We investigated the patterns and predictors of recurrence and survival in cryptogenic non-B, non-C, non-alcoholic hepatocellular carcinoma (CR-HCC). We compared the findings with those hepatitis virus B (B) and hepatitis virus C (C)-HCC. CR-HCC does not include HCC developed on NASH.

**Methods:**

From 1990 to 2011, of 676 patients who underwent primary curative liver resection as initial therapy for HCC at our institution, 167 had B-HCC, 401 had C-HCC, and 62 had CR-HCC. Differences between three groups were analyzed using the Chi-squared test. Cumulative overall survival (OS) and disease-free survival (DFS) were determined by the Kaplan-Meier method, prognostic factors involved in OS/DFS were evaluated by univariate analysis using the log-rank test, and stepwise Cox regression analysis.

**Results:**

Liver function was better in CR-HCC than in B/C-HCC, and mean tumor size was larger in CR-HCC than in B/C-HCC. In CR-HCC, OS was equivalent to that of B/C-HCC, and DFS was equivalent to that of B-HCC. Both tumor-related factors and background liver function appeared to be prognostic factors for three groups.

**Conclusion:**

Our findings indicate that the probability of survival of advanced CR-HCC was not longer than that of B/C-HCC. Given our findings, a postoperative follow-up protocol for CR-HCC should be established alongside that for B/C-HCC.

## Background

A nationwide follow-up survey by the Liver Cancer Study Group of Japan found that the incidence of hepatitis virus B(B)-related and that of hepatitis virus C(C)-related hepatocellular carcinoma(HCC) had decreased over the previous decade, possibly owing to the prevention of maternal-to-fetal transmission, careful screening of blood products, and the promotion of antiviral therapy [1]. However, the number of patients with HCC from other causes more than doubled over the same period, from 6.8 to 17.3 % [1]. Many hepatologists have noted a rapid increase in the proportion of cases of HCC negative for both hepatitis B surface antigen (HBsAg) and hepatitis C antibody (HCVAb), so-called “non-B non-C HCC” (NBNC-HCC), with incidence increasing steadily from 8.7 % in 2000 to 14.9 % in 2007 [2]. However, the definition of NBNC-HCC is still indistinct because it included HCC derived from a number of different etiologies such as alcoholic liver disease, nonalcoholic steatohepatitis (NASH), autoimmune hepatitis, primary biliary cirrhosis, and unknown causes. It is non-scientific to consider such a variety of diseases as one set. In this study, we focused on HCC derived from unknown causes, namely cryptogenic HCC (CR-HCC). Until recent, the patient and tumor characteristics and long-term postoperative prognosis of these patients has been still unknown because of their rarity. The aim of this study is to reveal the pre and postoperative characteristics of CR-HCC by comparing to the HCC derived from viral hepatitis.

## Methods

### Patients and definition of background etiology

From 1990 to 2011, 676 patients underwent primary curative liver resection as initial therapy for HCCs were retrospectively reviewed. The median follow-up period was 64.2 months (range; 5.6–233.8 months). The histopathological variables were defined according to the General Rule for the Clinical and Pathological Study of Primary Liver Cancer Study Group of Japan, and the pathological classification system of the World Health Organization [3, 4]. We defined the presence of vascular invasion and/or intrahepatic metastasis as cancer spread. The indications for hepatectomy were basically the same as those recommended in the Consensus-Based Clinical Practice Manual of the Japan Society of Hepatology [5].

In this study, patients with hepatitis B infection were defined as those who were seropositive for hepatitis B virus surface antigen, and patients with hepatitis C were defined as those who were seropositive for hepatitis C virus antibody. The 8 patients with co-infection of hepatitis B and C were excluded. Occult hepatitis B infection is defined as the existence of low-level HBV-DNA in the serum (<200 IU/mL), cells of the lymphatic (immune) system, and/or hepatic tissue in patients with serological markers of previous infection (anti-HBc and/or anti-HBs positive) and the absence of serum HBsAg [6]. Daily alcohol consumption was calculated from forms of alcohol and frequency. Alcoholic liver disease was defined as chronic liver injury with daily alcohol consumption ≧80 g/day without another definite etiology. NAFLD was defined as a history of fatty liver or who were diagnosed with fatty liver, radiologically or pathologically, with alcohol consumption < 20 g/day [7]. Autoimmune hepatitis (AIH) is a chronic disease of unknown cause, characterised by continuing hepatocellular inflammation and necrosis, which tends to progress to cirrhosis. Immune serum markers are often present and the disease is often associated with other autoimmune diseases [8, 9]. Primary biliary cirrhosis (PBC) is a chronic cholestatic liver disease with a predilection for the female gender, characterized by destruction of intrahepatic bile ducts that ultimately progresses to cirrhosis [10].

### The definition of cryptogenic HCC

The definition of CR-HCC was as follow; the HCC patients with unknown background liver etiology after exclusion of all other testable liver disease etiologies such as viral hepatitis including occult HBV infection, alcoholic liver damage, non-alcoholic steato-hepatitis (NASH), autoimmune hepatitis, primary biliary cirrhosis, severe steatosis, hemochromatosis, Wilson’s disease, and α1 antitrypsin deficiency.

### Study design

We compared the pre and postoperative patient’s characteristics, recurrence patterns, DFS, and OS between CR-HCC and HCC derived from viral hepatitis. The prognostic predictors of CR-HCC were investigated by multivariate analysis.

### Detection and definition of recurrence

Follow up of primary treatment was performed by tumor marker testing every month and ultrasonography (US) every 3 months during the first year. Thereafter, a new follow-up period was determined based on individual risk and likelihood of recurrence. Annual dynamic computed tomography (CT) and/or magnetic resonance imaging was performed when recurrence was suspected.

Tumor recurrence was suspected in the following circumstances: 1) progressive elevation of serum alpha-fetoprotein (AFP); 2) US detection of a new hepatic lesion; 3) contrast enhancement on CT during the arterial phase; or 4) high tumor vascularity (tumor stain) on hepatic angiography. Tumor recurrence, size, and number were determined on CT or CT angiography. Extrahepatic recurrence was determined on CT, magnetic resonance imaging, and scintigraphy. The site and pattern of initial recurrence was defined as follows: 1) marginal or same subsegment recurrence, 2) new subsegment recurrence, or 3) extrahepatic recurrence.

### Statistical analysis and ethics

Pairwise differences of proportions and means were analyzed using the Chi-squared test. Cumulative OS and DFS were analyzed by the Kaplan-Meier method. The prognostic factors involved in DFS and OS were evaluated using the log-rank test. In the multivariate analysis, variables associated with DFS and OS were identified by stepwise Cox proportional hazards models. Variables identified by simple Cox proportional hazards models were selected for potential association with recurrence based on previous studies or our clinical experience. The variables chosen were age, sex, number of tumors (single/multiple), type of hepatic resection (anatomical/non-anatomical), transfusion, degree of differentiation of the main tumor (well-moderate/poor), tumor size (<50/≥50 mm), metastasis, formation of tumor capsule (Fc), liver cirrhosis (LC), surgical margin (positive/negative), indocyanine green retention rate at 15 min (ICGR15; <15/≥15 %), platelet count (<10 × 10^4^/≥10 × 10^4^ platelets/ml), AFP (<100/≥100 AU/l) and des-r-carboxyprothrombin (DCP; <100/≥100 AU/l). Variables with significance of *p* < 0.15 in the simple Cox proportional hazards models were entered into multiple Cox proportional hazards models. In multiple Cox proportional hazards models, *p* < 0.05 was considered significant. All statistical analysis was performed using SPSS ver.19 (SPSS Inc., Chicago, IL). This study was approved by the Institutional Review Board of Toranomon hospital.

## Results

### Patient characteristics

Table 1 shows the profiles and data of all 630 patients. Some factors differed between the three groups. In the B-HCC group, the mean age was lower and men were more predominant than in the other two groups. The incidences of DM and hypertension in the patients with CR-HCC were higher than in the patients with both B-HCC and C-HCC (*P* < 0.001 and *P* < 0.001, respectively). Platelet count was higher in the CR-HCC group than in the C-HCC group. Regarding liver function, ICGR15 was highest in the C-HCC group and lowest in the B-HCC group, and the proportion of patients with liver cirrhosis was lowest in the CR-HCC group.

**Table 1:** Characteristic of patients with CR-HCC, B-HCC, and C-HCC

	CR-HCC (*n* = 62)	B-HCC (*n* = 167)	C-HCC (*n* = 401)	*p*-value CRvsB/ CRvsC/BvsC
Preoperative and operative factors				
Age (years)	68 (36–81)	54 (28–78)	64 (40–87)	<0.001/ 0.053/ <0.001
Gender (Male/Female)	46/16	144/23	287/114	0.028/ 0.397/ <0.001
BMI	22.7 (14.0–29.3)	22.9 (17.4–33.8)	22.8 (15.5–40.4)	0.879/ 0.951/ 0.692
Diabetes Mellitus	25 (40.3 %)	18 (10.7 %)	55 (13.7 %)	<0.001/ <0.001/0.341
Hypertension	24 (38.7 %)	16 (9.6 %)	58 (14.5 %)	<0.001/ <0.001/0.115
Plt	16.2 (4.6–32.9)	14.1 (1.5–30.1)	11.9 (3.1–32.9)	0.055/ 0.005/ 0.119
AFP	7.2 (1.6–23,530)	11 (0.8–66,810)	21.9 (1–137,000)	0.201/ 0.532/ 0.074
DCP	38 (0.07–23,676)	21 (0.07–10,200)	17 (0.07–18,703)	0.033/ <0.001/ 0.033
Anatomical resection	22 (35.5 %)	36 (21.6 %)	74 (18.5 %)	0.017/ 0.001/ 0.205
ICG	15.5 (3–68)	12 (3–53)	21 (3–96)	0.023/ 0.002/ <0.001
Child (A/B/C)	60/ 2/ 0	152 /15/ 0	357/ 44/ 0	0.431/ 0.218/ 0.102
MELD score (Median)	7 (6–12)	7 (6–14)	7 (6–14)	0.322/ 0.456/ 0.120
Pathological factors				
Tumor size (mm):greatest dimension	28 (9–114)	22 (6–120)	21 (2–100)	0.002/ <0.001/ 0.561
Poorly differentiated tumor	15 (24.2 %)	36 (21.6 %)	68 (17.0 %)	0.529/ 0.182/ 0.108
Multiple tumor	11 (17.7 %)	18 (10.8 %)	60 (15.0 %)	0.129/ 0.346/ 0.130
Cancer spread	20 (32.3 %)	39 (23.4 %)	86 (21.4 %)	0.153/ 0.066/ 0.346
LC	16 (25.8 %)	101 (60.5 %)	239 (59.6 %)	<0.001/ <0.001/ 0.405
Overall survival rate				
1 year	96.7 %	95.8 %	97.5 %	0.478/ 0.417/ 0.001
3 years	87.9 %	86.9 %	87.6 %	
5 years	75.0 %	81.6 %	74.7 %	
10 years	56.1 %	58.4 %	39.3 %	
Disease-free survival rate	28/62 (45.2 %)	99/167 (59.3 %)	274/401 (68.3 %)	0.575/ 0.026/ 0.004
1 year	81.4 %	79.3 %	81.9 %	
3 years	64.4 %	61.6 %	48.8 %	
5 years	50.4 %	48.6 %	34.0 %	
10 years	34.9 %	27.2 %	18.5 %	
Site and patterns of initial recurrence				
Marginal and same subsegment	3 (10.7 %)	14 (14.3 %)	52 (19.0 %)	
Other subsegment	20 (71.4 %)	80 (80.8 %)	213 (77.7 %)	
Extrahepatic recurrence.	5 (17.9 %)	5 (5.1 %)	9 (3.3 %)	

*CR-HCC* cryptogenic hepatocellular carcinoma, *B-HCC* hepatitis virus B-related hepatocellular carcinoma, *C-HCC* hepatitis virus C-related hepatocellular carcinoma, *AFP* alpha-fetoprotein, *DCP* des-r-carboxyprothrombin, *ICG* indocyanine green retention rate, *LC* Liver cirrhosis

With respect to operative factors and pathological factors, tumor size and proportion of anatomical resection were highest in the CR-HCC group. Serum DCP was highest in the CR-HCC group and lowest in the C-HCC group. Number of tumor, degree of differentiation, and proportion of metastatic cases showed no significant differences between the three groups (Table 1).

### Outcomes

**Fig. 1:**
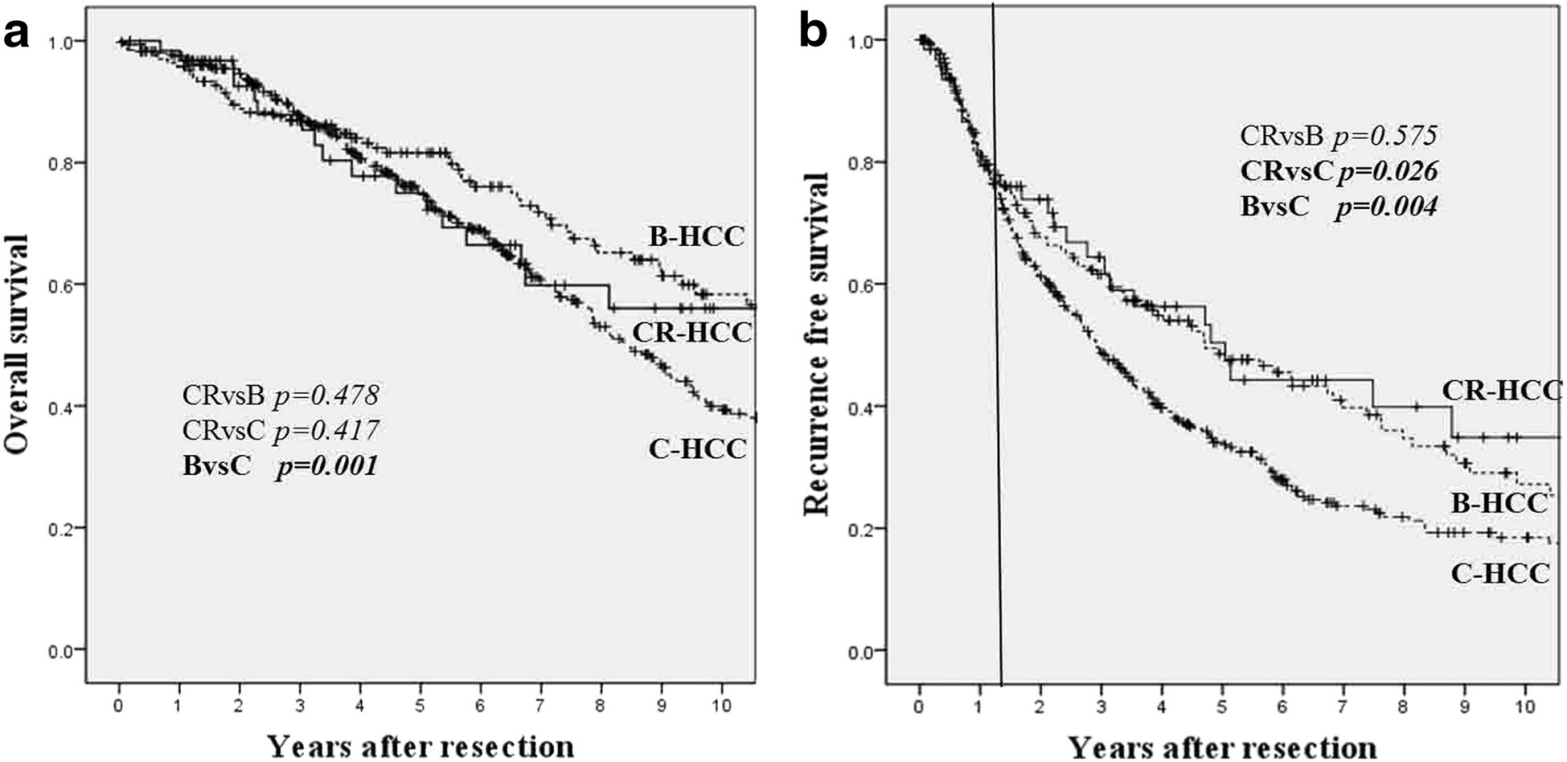
Overall survival (**a**) and recurrence-free survival (**b**) rates after primary curative resection of cryptogenic non-B, non-C, non-alcoholic hepatocellular carcinoma

OS was higher in the B-HCC group than in the C-HCC group (*p* = 0.001), while DFS was lowest in the C-HCC group. No differences in OS or DFS were found between the CR-HCC and B-HCC groups (Fig. 1). When assessing whether CR-HCC, B-HCC, and C-HCC were independent adverse prognostic factors, we found that C-HCC was an independent adverse prognostic factor for both OS and DFS (CR-HCC and B-HCC vs C-HCC OS: *p* = 0.001; DFS: *p* = 0.001). On the other hand, CR-HCC was not an independent adverse prognostic factor for either OS or DFS compared with B-HCC and C-HCC (CR-HCC vs B-HCC and C-HCC OS: *p* = 0.119; DFS: *p* = 0.773).

Over a median observation period of 5.35 years, there were 28 recurrences in the CR-HCC group (45.2 %), 99 in the B-HCC group (59.3 %), and 274 in the C-HCC group (68.3 %). DFS time was defined as the interval between the day of surgery and diagnosis of recurrence. The sites and patterns of initial recurrence in the CR-HCC, B-HCC, and C-HCC groups are shown in Table 1. The recurrence sites showed no significant differences between the three groups. However, the number of recurring tumors was different. In total, liver recurrence was observed in 23 patients with CR-HCC (single 13 (56.5 %)/multiple 10 (43.5 %)), 94 patients with B-HCC (single 60 (63.8 %)/multiple (34 (36.2 %)), and 265 patients with C-HCC (single 52 (19.6 %)/multiple 213 (80.4 %)).

### Prognostic factors for OS and DFS

The prognostic factors for OS and DFS in the three groups are shown in Tables 2 and 3. In the CR-HCC group, multiple tumors, poorly differentiated carcinomas, large tumors, and low platelet counts were independent prognostic factors for OS. Large tumors, multiple tumors, and low platelet counts were independent prognostic factors for recurrence.

**Table 2:** Results of univariate and multivariate analysis of predictors for the recurrence of CR-HCC, B-HCC, and C-HCC

Variables	Category	*P(uni)*	*P(multi)*	Multi hazard ratio
CR-HCC				
Number of tumors	Single/multiple	0.004		
Intraoperative RBC transfusion	Yes/no	0.060		
Degree of primary differentiation	well, moderate/poorly	0.078		
Tumor size	<50/≧50 mm	0.029	0.014	3.0 (1.3–7.0)
Cancer spread	Yes/no	0.005	0.001	4.1 (1.7–9.6)
Platelet count	<10 × 10^4^/≧10 × 10^4^ /ml	0.058	0.004	4.4 (1.6–12.1)
B-HCC				
Degree of differentiation of the main tumor	well, moderate/poorly	0.140		
Tumor size	<50/≧50 mm	0.018		
Platelet count	<10 × 10^4^/≧10 × 10^4^ /ml	0.070	0.038	1.7 (1.0–2.7)
C-HCC				
Number of tumors	Single/multiple	0.004	0.016	1.5 (1.1–2.2)
Degree of differentiation of the main tumor	well, moderate/poorly	0.015	0.067	1.3 (0.98–1.9)
Tumor size	<50/≧50 mm	0.067		
Cancer spread	Yes/no	0.001	0.001	1.6 (1.2–2.2)
Surgical margin	Positive/Negative	0.116		
LC	Yes/no	0.004	0.058	1.3 (0.99–1.7)
ICGR15	<15/≧15 %	0.003	0.014	1.5 (1.1–2.0)
Platelet count	<10 × 10^4^/≧10 × 10^4^ /ml	0.014		
AFP	<100/≧100 AU/l	<0.001	0.038	1.4 (1.0–1.8)

*CR-HCC* cryptogenic hepatocellular carcinoma, *B-HCC* hepatitis virus B-related hepatocellular carcinoma, *C-HCC* hepatitis virus C-related hepatocellular carcinoma, *AFP* alpha-fetoprotein, *DCP* des-r-carboxyprothrombin, *ICGR15* indocyanine green retention rate at 15 min, *LC* Liver cirrhosis

**Table 3:** Results of univariate and multivariate analysis of the predictors for overall survival in CR-HCC, B-HCC, and C-HCC

Variables	Category	*P(uni)*	*P(multi)*	Multi hazard ratio
CR-HCC				
Number of tumors	Single/multiple	0.062	0.024	3.9 (1.2–12.8)
Intraoperative RBC transfusion	Yes/no	0.004	0.095	4.0 (0.79–20.3)
Degree of differentiation of the main tumor	well, moderate/poorly	0.019	0.001	8.5 (2.3–31.3)
Tumor size	<50/≧50 mm	0.040	0.042	5.3 (1.1–26.0)
LC	Yes/no	0.068		
Platelet count	<10 × 10^4^/≧10 × 10^4^ /ml	0.004	<0.001	9.6 (2.7–34.3)
B-HCC				
Number of tumors	Single/multiple	0.055		
Intraoperative RBC transfusion	Yes/no	0.004	<0.001	13.4 (4.7–38.1)
Degree of differentiation of the main tumor	well, moderate/poorly	0.007	0.003	2.7 (1.4–5.2)
Tumor size	<50/≧50 mm	0.004		
LC	Yes/no	0.041		
ICGR15	<15/≧15 %	<0.001	<0.001	3.5 (1.8–6.8)
C-HCC				
Gender	Male/Female	0.626	0.012	1.6 (1.1–2.4)
Number of tumors	Single/multiple	0.101		
Intraoperative RBC transfusion	Yes/no	<0.001	0.016	1.9 (1.1–3.1)
Cancer spread	Yes/no	0.003	0.002	1.7 (1.2–2.3)
LC	Yes/no	<0.001	<0.001	2.1 (1.5–3.0)
ICGR15	<15/≧15 %	<0.001	0.003	1.9 (1.2–2.8)
Platelet count	<10 × 10^4^/≧10 × 10^4^ /ml	0.001		
AFP	<100/≧100 AU/l	0.005	0.018	1.5 (1.1–2.1)

*CR-HCC* cryptogenic hepatocellular carcinoma, *B-HCC* hepatitis virus B-related hepatocellular carcinoma, *C-HCC* hepatitis virus C-related hepatocellular carcinoma, *AFP* alpha-fetoprotein, *DCP* des-r-carboxyprothrombin, *ICGR15* indocyanine green retention rate at 15 min, *LC* Liver cirrhosis

In the B-HCC group, poorly differentiated carcinomas, transfusion, and high ICGR15 levels were independent prognostic factors for OS. Only low platelet count was an independent prognostic factor for recurrence.

In the C-HCC group, being male, transfusion, metastasis, high ICGR15 levels, LC, and high AFP levels were independent prognostic factors for OS. Multiple tumors, metastasis, high ICGR levels (≥15 %) and high AFP levels (≥100 AU/l) were independent prognostic factors for recurrence. LC and poorly differentiated carcinoma are potential prognostic factors but did not reach statistical significance.

## Discussion

There have been no health outcomes studies focusing on CR-HCC patients as the research subjects. Here we investigated OS, DFS, recurrence patterns, and prognostic factors in 62 such patients. Our results indicate that the need for postoperative follow up is as relevant for CR-HCC as it is for B-HCC and C-HCC.

First, comparing the clinical backgrounds between the three patient groups, the proportion of patients with LC was lowest in the CR-HCC group, which therefore had the highest preoperative platelet count. In the liver function category, the parameters reflecting the incidence and severity of chronic hepatitis or LC were greatest in the C-HCC group, followed by the B-HCC group and then the CR-HCC group. Among the three groups, liver function was best in the CR-HCC group. Typically, B-HCC and C-HCC patients with chronic hepatitis are followed periodically, whereas NBNC-HCC tends to be discovered at an advanced stage because the disease derives from normal liver. In this study, the mean tumor size in CR-HCC cases (28 mm) was larger than that in B-HCC (22 mm) and C-HCC (21 mm) cases. The smaller tumors in the latter groups may be because of the periodic screening for HCC these patients receive. DCP levels were found to correlate with tumor size. Generally speaking, the larger the tumor, the higher the serum DCP levels. In this study, the CR-HCC patients had larger tumors and higher preoperative serum DCP levels than the B-HCC and C-HCC patients. We chose anatomical resection for CR-HCC patients in many cases because their liver function was better than that of patients in the other two groups and because their tumors were larger. It is generally considered that anatomical resection will benefit survival and reduce recurrence.

Nanashima et al. [11] and Kondo et al. [12] reported higher DFS and OS rates in NBNC-HCC patients than in B-HCC and C-HCC patients. However, our analysis showed no significant differences in OS between CR-HCC, B-HCC, and C-HCC patients. A possible reason for this lack of differences is that cases of CR-HCC in our study tended to be discovered at the advanced stage, perhaps because the patients with this disease had normal liver function or did not attend periodic medical examinations. On the other hand, detection of the smaller tumors in the B-HCC/C-HCC group may have been found during periodic screening for HCC. Therefore, the probability of survival is not necessarily good in patients with advanced-stage CR-HCC compared with B-HCC/ C-HCC patients. As shown in Fig. 1, the OS curve for CR-HCC patients showed signs of leveling off around 7 to 8 years, whereas the curves for the other two groups continued to decrease even after 10 years. As surgical techniques and perioperative management have improved, so have the postoperative outcomes of hepatic resection for HCC. However, the high rate of recurrence in HCC patients who have undergone hepatic resection remains a serious problem.

At first, the recurrence site showed no significant differences between the three groups. However, the number of initially recurring tumors was different, and multiple liver recurrences were higher in the C-HCC group than in the other two groups. In this study, DFS was lower in the C-HCC group than in the other two groups, and no differences were observed between the CR-HCC and B-HCC groups. As shown in Fig. 1, the analysis of the CR-HCC DFS curve shows a point of inflection around 1 year after resection. The slope of the curve is similar in all three groups until 1 year after resection, and thereafter the slope of the curves for CR-HCC and B-HCC decreases more than that for C-HCC. The DFS curves for CR-HCC and B-HCC are almost the same. Our results are in agreement with those of Cucchetti et al. who observed a point of inflection for viral HCC 2 years after surgery. This time point was taken to distinguish “early recurrence” (<2 years) and “late recurrence” (>2 years). Recurrence after resection occurs in the remnant liver as a result of intrahepatic metastasis from the primary tumor or multicentric carcinogenesis (MC) [13]. The novel and interesting aspect of our results is that the CR-HCC DFS curve showed a point of inflection around 1 to 2 years after resection, which was almost the same as the B-HCC curve. Eguchi et al.[14] reported that after curative liver resection, recurrence within 2 years occurred most often in the form of intrahepatic metastasis through vascular invasion, whereas recurrence occurring 2 years after R0 resection was most often MC and a different clone from the first resected HCC. Cucchetti et al. [13] also reported that multinodular intrahepatic recurrence was more frequently observed in cases of early recurrence than in those of late recurrence. MC is considered the major cause of late recurrence in B-HCC and C-HCC patients [13, 14].

No significant differences in early recurrence were found between the three groups. On the other hand, significant differences in late recurrence were found between the three groups. Improved DFS was observed in the CR-HCC group compared with the C-HCC group due to a low incidence of MC caused by a chronic viral attack. However, comparison of the DFS curves between the CR-HCC and B-HCC groups revealed the same tendency and no statistically significant differences between them.

Turning now to prognostic factors, we found many differences in these factors between our three groups. The prognostic factors for postoperative recurrence of B-HCC and C-HCC have been extensively studied, and a postoperative follow-up protocol for both is currently being established. However, the literature contains no reports on the prognostic factors for postoperative recurrence and OS in CR-HCC. Given the findings of some previous studies [5, 11, 15–20], we predicted for our study that only the prognostic factors for CR-HCC would be tumor factors and that background liver function would not be prognostic because the liver function of CR-HCC patients is better than that of B-HCC/C-HCC patients. Unexpectedly, however, we found that background liver function was also a prognostic factor for CR-HCC as well as B-HCC/C-HCC. Therefore, both tumor-related factors and background liver function appear to be prognostic factors for all three types of disease.

Although there was no apparent background liver disease in the CR-HCC patients, no differences in OS and DFS were found between them and the B-HCC patients, nor was there a difference in DFS between them and the C-HCC patients. Given our findings, a postoperative follow-up protocol for NBNC-HCC should be established alongside that for B-HCC/C-HCC.

The major limitation of our study was enrollment. Although the number of NBNC-HCC patients has been increasing recently, the proportion of NBNC-HCC cases among all HCC cases is still only 15.0 % at most. In addition, CR-HCC with LC may include burnt-out NASH. If we can accumulate more cases, a more precise analysis might show strong similarities in background liver function in NBNC-HCC.

## Conclusion

Although CR-HCC patients did not have clear background liver disease, their OS was equivalent to that of B-HCC and C-HCC patients, and DFS was equivalent to that of B-HCC patients. Tumor-related factors and background liver function were prognostic factors in all three groups. For now, we recommend that when patients with NBNC-HCC have risk factors for postoperative recurrence such as large tumors (≥50 mm), multiple tumors, and low platelet counts, they should be periodically followed according to the same follow-up protocol as if they had B-HCC or C-HCC.

## Abbreviations

CR-HCC: Cryptogenic hepatocellular carcinoma
B-HCC: Hepatitis virus B-related hepatocellular carcinoma
C-HCC: Hepatitis virus C-related hepatocellular carcinoma
NASH: Nonalcoholic steatohepatitis
AIH: Autoimmune hepatitis
PBC: Primary biliary cirrhosis
AFP: Alpha-fetoprotein
DCP: des-r-carboxyprothrombin
ICG: Indocyanine green retention rate
LC: Liver cirrhosis
